# Editorial: Model organisms and experimental models: opportunities and challenges in clinical and translational physiology

**DOI:** 10.3389/fphys.2023.1267842

**Published:** 2023-09-11

**Authors:** Jaromir Myslivecek, Chi-Wen Lung, Tania Martins-Marques, Samuel T. Orange, Mikio Hiura, Yonghe Ding

**Affiliations:** ^1^ First Faculty of Medicine, Charles University, Prague, Czechia; ^2^ Department of Kinesiology and Community Health, University of Illinois at Urbana-Champaign, Champaign, IL, United States; ^3^ Institute for Clinical and Biomedical Research, Faculty of Medicine, University of Coimbra, Coimbra, Portugal; ^4^ Newcastle University Centre for Cancer, Newcastle University, Newcastle upon Tyne, United Kingdom; ^5^ School of Biomedical, Nutritional and Sport Sciences, Faculty of Medical Sciences, Newcastle University, Newcastle upon Tyne, United Kingdom; ^6^ Center for Brain and Health Sciences, Aomori University, Aomori, Japan; ^7^ Department of Biochemistry and Molecular Biology, Mayo Clinic, Rochester, MN, United States; ^8^ The Biomedical Sciences Institute, Qingdao University, Qingdao, China

**Keywords:** porcine (pig) model, transgenic mice, zebrafish, trauma research model, sepsis, arteriovenous fistula, kidney cystic diseases, preterm birth

Exploring animals has a long-lasting history. The pre-historic man observed not only animal behavior to gain food but also needed knowledge of circannual rhythms to know animals’ paths and/or behavior in different year seasons.

Another key wisdom was knowledge of animal anatomy (Kinter et al., 2021). Gradually, in history, the preparation of treating substances (with animal components), the usage of animals to develop surgical procedures, and the first experiments (manipulation of living animals) by Aristotle (4th century BCE) appeared (Kinter et al., 2021). In the modern era, experimentation began in the 18th century and was highly unrolled in the 19th and 20th centuries. However, the number of animals used in experiments gradually decreased from the end of the nineties to the 20th century (Kinter et al., 2021).

This Research Topic is focused on opportunities and challenges in clinical and translational physiology when using model organisms and animal models. A wide spectrum of models were used in original research studies or reviewed: pigs, small animal models (mainly mice and also transgenic models), and zebrafish. Although it seems that porcine models in translational physiology are the most used and more clinically relevant alternative to small animal models, the convenience of genetic manipulation on specific genes made models with this option highly relevant to these purposes.

In the first paper on this Research Topic, Gihring et al. described trauma models using mice. In this mini review, they emphasize the importance of the development of a clinically relevant murine model. The obvious conflict exists between simulating clinically relevant situations and elucidating molecular mechanisms. Similarly, the researchers can use two models of trauma: clinically and mechanistically relevant, which are deeply discussed in this review. The authors also describe the advantages and disadvantages of established mouse trauma models developed to simulate clinically relevant situations, and, finally, summarize the established mouse models in the field of trauma research developed to simulate clinically relevant situations. The next paper, a review by Vintrych et al., is focused on sepsis and modeling this pathological life-challenging situation. The authors compare large and small animal models and conclude that the large animal (porcine) models represent a more clinically relevant alternative to small animal models, mainly when searching for therapeutic strategies. They also emphasize that the findings obtained in small animal (transgenic) models should be verified in clinically relevant large animal models before translation to the clinical level. Original research by Valerianova et al. focused, in the porcine model, on comparing the hemodynamic effect of a large arteriovenous fistula in different cardiac output states (high and low), considering changes in arterial blood pressure (systemic vasoconstriction). In conclusion, the experiments, performed by this group, stress the detrimental role of a large arteriovenous fistula on hemodynamics in models simulating the critical conditions. The next paper by Koslow et al. focused on cystic kidney disease and provided evidence to support zebrafish as a conserved animal model for genetic analysis. As there were concerns about the suitability of zebrafish as a model organism for cystic kidney disease, the authors found 81 corresponding zebrafish homologs of 82 human cystic kidney disease genes. Further gene analysis revealed that there exists remarkable molecular conservation, supporting zebrafish as a useful animal model for genetic study of cystic kidney disease. The last paper of this Research Topic, a review by Sun et al. describes multiple animal models of preterm birth (mice, rats, rabbits, sheep, and pigs), as privileged tools to study the occurrence of preterm birth and to evaluate potential therapeutic interventions in human newborns. The physiological features of growth and development of preterm pigs at different gestational ages were further detailed, with the porcine model being found as suitable for nutritional fortification, necrotizing enterocolitis, neonatal encephalopathy and hypothermia intervention, mechanical ventilation, and oxygen therapy in preterm infants.

In conclusion, not only large animal models are appropriate in translational and clinical physiology. Some aspects are better to be simulated using small/transgenic animals and, as demonstrated by Koslow et al., lower vertebrates can be a useful animal model for genetic study of cystic kidney disease.
